# Comparison of clinical outcomes between femtosecond laser-assisted versus conventional phacoemulsification

**DOI:** 10.1186/s40662-018-0102-5

**Published:** 2018-04-23

**Authors:** Robert Edward Ty Ang, Michelle Marie Salcedo Quinto, Emerson Marquez Cruz, Mark Christian Reyes Rivera, Gladness Henna Austria Martinez

**Affiliations:** 1 Asian Eye Institute, Rockwell Center, Makati City, 1200 Philippines; 2 Cardinal Santos Medical Center, 10 Wilson St., San Juan City, 1502 Philippines

**Keywords:** Femtosecond laser, Phacoemulsification, Cataract, Cumulative dissipated energy, Endothelial cell loss, Flare

## Abstract

**Background:**

To compare femtosecond laser-assisted versus conventional phacoemulsification in terms of visual and refractive outcomes, cumulative dissipated energy, anterior chamber inflammation and endothelial cell loss.

**Methods:**

In this retrospective cohort study, records of eyes that underwent femtosecond laser-assisted cataract surgery (FLACS) or conventional phacoemulsification (CP) were reviewed. The Victus femtosecond laser (Bausch and Lomb, Germany) was used to carry out corneal incisions, anterior capsulotomy, and lens fragmentation in FLACS procedures. Manifest refraction spherical equivalence (MRSE), uncorrected distance visual acuity (UDVA), corrected distance visual acuity (CDVA), cumulative dissipated energy (CDE), postoperative cells and flare and endothelial cell count data were collected. Subgroup analysis of the visual acuity tests was performed based on the type of intraocular lens implanted (monofocal, monofocal toric, multifocal, multifocal toric, accommodating).

**Results:**

A total of 735 eyes were included in the study (296 eyes for the FLACS group and 439 eyes for the CP group). At one year follow-up, 120 eyes comprised the FLACS group and 265 eyes for the CP group. MRSE in the FLACS group was − 0.16 ± 0.58 D and − 0.20 ± 0.52 D in the CP group (*P* = 0.50). UDVA in the FLACS group was 20/25 (mean logMAR 0.12 ± 0.13) and 20/25 (mean logMAR 0.11 ± 0.13) in the CP group (*P* = 0.48). CDVA was 20/20 (mean logMAR 0.03 ± 0.07) in the FLACS group and 20/20 (mean logMAR 0.02 ± 0.06) in the CP group (*P* = 0.15). No statistically significant trend was seen for FLACS versus CP by intraocular type for visual acuity. CDE for the different cataract grades ranged from 6.97 ± 5.74 to 29.02 ± 16.07 in the FLACS group and 7.59 ± 6.42 to 35.69 ± 18.30 in the CP group. The FLACS group was significantly lower for post-operative central corneal edema (*P* = 0.05), cells and flare (*P* = 0.01), and endothelial cell loss (*P* = 0.04).

**Conclusions:**

Femtosecond laser-assisted cataract surgery and conventional phacoemulsification had similar refractive and visual outcomes. Phacoemulsification energy, anterior chamber inflammation and corneal endothelial cell loss were less in the femtosecond laser group.

## Background

The most commonly performed ophthalmologic procedure in the world is cataract surgery, with approximately 20 million surgeries done in 2010 and estimated to reach 32 million by 2020 [1]. Phacoemulsification is the current preferred method of removing cataracts wherein the lens material of the cataract is softened using ultrasonic energy (emulsify) followed by extraction from the eye through irrigation and suction [2]. Specific steps in conventional cataract surgery through phacoemulsification include creating corneal incisions using a blade or keratome, manually opening the anterior capsule (capsulotomy) using a forceps or bent needle, fragmenting the cataractous lens with ultrasonic energy and chopper instruments, suction of lens material, implantation of an intraocular lens (IOL) and finally aspiration and clean-up of viscoelastic and retained lens cortical fragments [2]. In recent years, the femtosecond laser has been utilized to perform the vital steps of corneal incision, anterior capsulotomy and lens fragmentation [2].

A femtosecond laser is an infrared laser that works by photodisruption wherein laser energy absorbed by the tissue induces rapid expansion, creating microcavitation bubbles and acoustic shock waves that cause morphological changes [3]. It acts like a cutting tool by firing thousands of bubbles side by side thereby creating a plane of dissection. The near infrared wavelength of 1053 nm is not absorbed by optically clear tissues such as the cornea and affect only the tissue at the focus point of the beam [3]. The reduced pulse duration in the femtosecond range (10^− 15^ s) compared with Nd:YAG (10^− 9^ s) means significant reduction of collateral tissue damage [2, 3].

Femtosecond laser technology has been initially introduced as a bladeless option of corneal flap creation in laser in situ keratomileusis (LASIK) [4, 5]. The predictability, accuracy and lower frequency of complications in corneal flap creation afforded by femtosecond lasers and the introduction of premium IOLs paved the way of incorporating this type of laser to ‘pre-treat’ cataractous eyes to produce more precise and reproducible clear corneal incisions and capsulotomies and placement of lens softening patterns prior to phacoemulsification [6]. It was postulated that since the femtosecond laser can do a perfect, round-shaped, smaller capsulorhexis, it can hold the lens better and prevent intraocular lens rotation, tilting, and decentration. This may prove advantageous more so for multifocal, toric or accommodating intraocular lenses [7]. Currently, four commercial femtosecond laser-assisted cataract surgery platforms are available: LenSx (Alcon, USA), Catalys (AMO, USA), LensAR (Lensar, USA), Victus (Bausch and Lomb, Germany) and Femto LDV 28 (Ziemer, Switzerland). These platforms vary slightly in their docking system and imaging modality, but the overall procedure performed is similar.

Studies have shown that femtosecond laser-assisted cataract surgery (FLACS) produce better clear corneal incision morphology [8], more precise and reproducible capsulotomies [9–13], better IOL centration [11], better predictability of IOL power calculation [12, 13], significant reduction in effective phacoemulsification time [9, 14–16], reduced ultrasound power and ultrasound time [16], less post-operative anterior segment inflammation [17], significant reduction in early post-operative corneal edema [18], decreased central endothelial cell loss [18, 19], and faster visual recovery and earlier stabilization of refraction [13]. Even with these reported benefits, issues of additional learning curve [20–22] for surgeons and costs [23] have prevented surgeons and eye centers worldwide from fully converting to FLACS. Adverse effects associated with femtosecond laser-assisted cataract surgery that have likewise hindered universal adaption include endothelial cell loss for laser-automated corneal incisions [18], capsulotomy margin irregularity [23, 24], anterior capsule tears [10, 25–28], miosis [29, 30], increase in intraocular pressure [31–33], and cystoid macular edema [34].

Additional studies on femtosecond laser-assisted cataract surgery need to be performed to determine the impact of femtosecond lasers on patient outcomes and quality of cataract surgery. In this study, we analyzed patients from a single center who underwent either FLACS or conventional phacoemulsification (CP) and compared their visual and refractive outcomes, cumulative dissipated energy, anterior chamber inflammation and endothelial cell loss.

## Methods

### Patients

This observational retrospective cohort study reviewed 735 patient records of FLACS and CP cases performed by a single surgeon at the Asian Eye Institute from January 2011 to September 2015. Approval by the institutional ethics committee was obtained and the study was performed according to the tenets of the Declaration of Helsinki. All patients 18 years and older with at least 1 month follow-up were included. Advancing age may be linked to more eye diseases, however, the study results should only be attributed to the removal of cataract hence other eye pathologies were excluded. Eyes with corneal scarring, keratoconus, amblyopia, glaucoma, retinal problems, macular pathology, and optic nerve damage were excluded to manage confounders and bias.

### Pre-operative assessment

Each patient had a complete ophthalmologic evaluation, which included anterior segment and dilated posterior segment examinations. Uncorrected distance visual acuity (UDVA), uncorrected near visual acuity (UNVA) using Jaegers Chart at 16 in., corrected distance visual acuity (CDVA), distance-corrected near visual acuity (DCNVA) at 16 in., refraction with sphere, cylinder and add, and manifest refraction spherical equivalent were logged. Other parameters noted were cataract grade based on the Lens Opacities Classification System III (LOCS III), average keratometry based on biometry (IOLMaster V.5, Carl Zeiss Meditech Inc., Jena, Germany), type of IOL used (monofocal, monofocal toric, multifocal, multifocal toric, accommodating), and endothelial cell density by specular microscopy (Konan CellChek XL, Konan Medical, Irvine, CA). Eyes were dilated with tropicamide 0.5%, phenylephrine 0.5% (Mydfrin, Santen, Japan), and ketorolac (Acular, Allergan, USA) 1 drop every 15 min for 3 cycles prior to surgery.

### Surgical procedure

Cataract surgery was performed on all patients under intravenous Midazolam 0.1 mg/ml and Fentanyl 0.5–1 mcg/kg/dose sedation and topical anesthesia. A 15-degree blade was used to create two paracentesis incisions at the 12:00 and 6:00 area, intracameral 75% lidocaine in fortified Balance Salt Solution (BSS Plus, Alcon, USA) and viscoelastic (Discovisc, Alcon, USA) was injected into the anterior chamber and a temporal clear cornea incision was created using a 2.75 mm keratome. Continuous curvilinear capsulorhexis was performed manually, followed by phacoemulsification with Infiniti (Alcon, Fort Worth, Texas, USA) using divide and conquer technique, bimanual irrigation and aspiration of cortical remnants, IOL implantation and evacuation of viscoelastic.

**Fig. 1:**
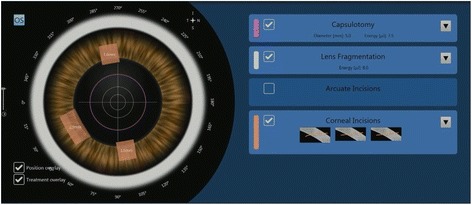
Femtosecond laser treatment planning for capsulotomy, lens fragmentation and corneal incisions

**Fig. 2:**
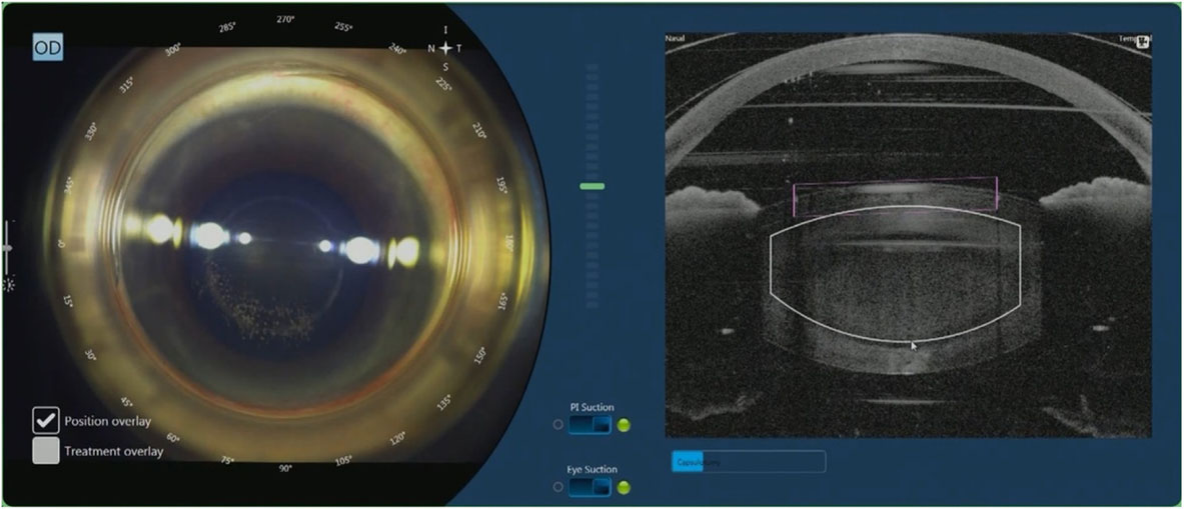
Femtosecond laser creating an anterior capsulotomy

Patients who opted for FLACS underwent the laser portion of the procedure first. The Victus (Bausch and Lomb, Germany) femtosecond laser was used to perform anterior capsulotomy, lens fragmentation then corneal incisions (temporal main incision and 2 paracentesis side ports), in that sequence (Fig. 1). A suction ring was first applied, after which a curved patient interface was docked over the cornea without flattening the corneal curvature. A round capsulotomy was created with energy set at 7.5 μJ (Fig. 2). The spot size was 3 μm with spacing of 6 μm along the path (Rim spot distance) and 4 μm between paths in the vertical cut direction (Rim path distance). The surgeon decided on the diameter of the capsulotomy based on the type of IOL to be used wherein 5.0 mm was selected when using C-loop lenses and plate haptic lenses (AT Lisa, Carl Zeiss, Germany) or 5.5 mm when using accommodating lenses with hinged haptics (Crystalens, Bausch and Lomb, USA).

**Fig. 3:**
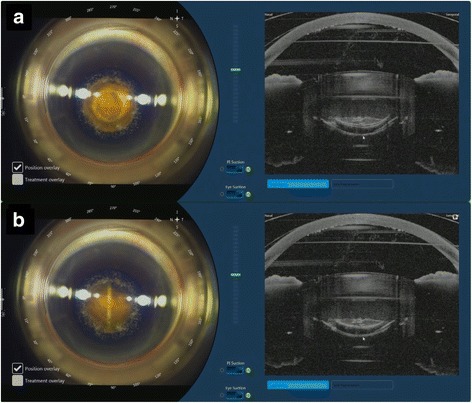
Femtosecond laser treatment lens fragmentation. **a** Circular pattern. **b** Radial pattern

**Fig. 4:**
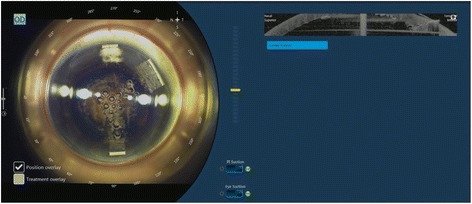
Femtosecond laser treatment creating a main port and two side port incisions

A combined circular (2 circles, diameter of 2.0 mm and 2.8 mm) and 4-cut cross-shaped pattern (diameter of 8.0 mm) was used for lens fragmentation with pulse energy at 8.0 μJ (Fig. 3). A 700 μm clearance zone was applied to avoid damaging the anterior and posterior capsule. Femtosecond laser was used to create two paracenteses and a clear corneal incision (Fig. 4). Width of the main port corneal incision was at 2.75 mm and 1.6 mm for each of the 2 side ports. Main port was positioned at 210^°^ for the right eye and 30^°^ for the left eye. Side ports were at 95^°^ and 275^°^ for the right eye, and at 100^°^ and 280^°^ for the left eye. Femtosecond laser treatment complications such as non-free floating capsulotomies, capsular tears, and suction breaks were tallied.

**Fig. 5:**
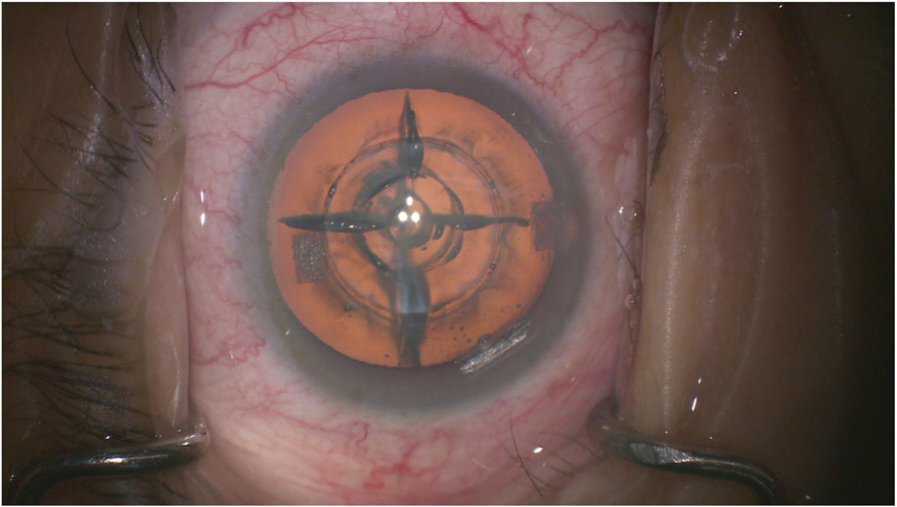
Post-femtosecond laser treatment showing anterior capsulotomy, lens fragmentation pattern and corneal incisions

**Fig. 6:**
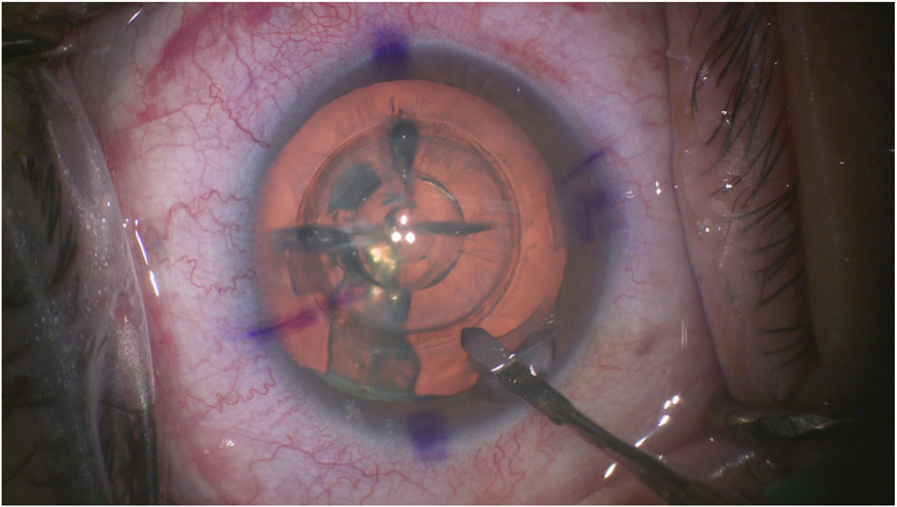
Corneal incision dissection using a blunt corneal femtodissector instrument

After treatment with the Victus femtosecond laser, patients were transferred to the operating theater for phacoemulsification (Fig. 5). After sedation, corneal incisions were opened using a designated instrument (Ang Corneal Femtodissector, Storz, USA) (Fig. 6) and the separated anterior capsule was removed with a forcep. All other steps were similar to conventional phacoemulsification.

IOLs used included the following (i) monofocal: Alcon Acrysof IQ, Bausch & Lomb Akreos Adapt AO, Bausch and Lomb Envista MX60, Zeiss CT Asphina; (ii) monofocal toric: Alcon Acrysof IQ Toric, Bausch & Lomb Envista MX60 Toric; (iii) multifocal: Alcon Acrysof IQ Restor, FineVision Physiol Trifocal, Oculentis Lentis, Zeiss AT Lisa Tri; and (iv) accommodating: Bausch & Lomb Crystalens. Cumulative dissipated energy displayed on the phacoemulsification machine were logged for both FLACS and CP based on cataract grade. The standardized test of Lens Opacities Classification System III (LOCS III) was used for grading the cataracts to lessen bias. Post-operative antibiotic, steroid and nonsteroidal anti-inflammatory drops were given to all patients.

Routine postoperative follow-up data scheduled at 1 day, 1 week, 1 month, 3 months, 6 months, and 1 year were collected. Variables included MRSE, UDVA, and CDVA for all IOLs and per IOL type. UNVA and DCNVA were analyzed for multifocal lenses and accommodating lenses. Central corneal edema, peripheral corneal edema, and aqueous cells and flare grading were recorded at 1 day and 1 week post-operation. Aqueous cells and flare were graded using the standardized test called the Standardization of Uveitis Nomenclature (SUN) Working Group Classification for anterior chamber inflammation to remove bias. Endothelial cell densities were recorded pre-operatively and at 1 month post-operation by specular microscopy (Konan CellChek XL, Konan Medical, Irvine, CA).

### Statistical analysis

Continuous data were summarized by mean ± standard deviation and categorical data by number (percentage). Comparisons among the FLACS and CP groups were analyzed using t-test for continuous variables with two categories, using a two-sided confidence interval of 95%. Chi-square test was used for an association between variables with more than 2 values. Statistical analyses were performed with SPSS 20.0 software (SPSS Inc., Chicago, Illinois). The level of significance was set at *P* value of less than or equal to 0.05 across all parameters.

## Results

This study evaluated 735 eyes, with 296 in the FLACS group and 439 in the CP group. The FLACS group had a higher age population, worse pre-operative DCNVA, higher pre-operative near vision add, and a higher incidence of grade 4 cataracts. The CP group had more grade 2 cataracts. The rest of the pre-operative parameters were similar (Table 1). Mean follow-up time for FLACS was 27.31 weeks and for CP, 33.94 weeks.

**Table 1:** Baseline Demographics

	No. of patients (%) or Mean ± SD		*P* Value
	FLACS (*n* = 296)	CP (*n* = 439)	
Age (years)	67.48 ± 10.33	65.21 ± 10.87	
Gender			
Female	182 (61.50%)	175 (61.40%)	0.99
Male	114 (38.50%)	110 (38.60%)	
Visual Acuity (logMAR)			
UDVA	0.78 ± 0.58 (20/125)	0.74 ± 0.54 (20/100)	0.32
UNVA	0.50 ± 0.26 (J8)	0.52 ± 0.25 (J8)	0.28
CDVA	0.25 ± 0.35 (20/32)	0.21 ± 0.31 (20/32)	0.06
DCNVA	0.46 ± 0.21 (J8)	0.37 ± 0.22 (J6)	0.00^*^
Refraction (D)			
Sphere	−0.77 ± 3.70	− 0.28 ± 3.82	0.10
Cylinder	−1.23 ± 0.82	− 1.22 ± 2.57	0.95
Add	2.55 ± 0.46	2.32 ± 0.68	0.00^*^
MRSE	− 1.36 ± 3.70	− 0.86 ± 3.98	0.09
LOCS III Cataract Grade			
NO1	25 (8.45%)	36 (8.20%)	0.04^*^
NO2	136 (45.95%)	239 (54.44%)	
NO3	79 (26.69%)	111 (25.28%)	
NO4	56 (18.92%)	53 (12.07%)	
Average Keratometry (D)	43.97 ± 1.72	44.20 ± 1.69	0.14
Type of Intraocular Lens			
Monofocal	62 (20.95%)	77 (17.54%)	
Monofocal Toric	59 (19.93%)	66 (15.03%)	
Multifocal	81 (27.36%)	107 (24.37%)	
Multifocal Toric	53 (17.90%)	70 (15.95%)	
Accommodating	41 (13.85%)	119 (27.11%)	

*CDVA =* corrected distance visual acuity; *CP =* conventional phacoemulsification; *D =* diopter; *DCNVA =* distance-corrected near visual acuity, *FLACS =* femtosecond laser-assisted cataract surgery; *LOCS III* = Lens Opacities Classification System III; *logMAR* = logarithm of the minimum angle of resolution; *MRSE =* manifest refraction spherical equivalent; *NO =* nuclear opalescence; *SD* = standard deviation; *UDVA =* uncorrected distance visual acuity; *UNVA =* uncorrected near visual acuity

^*^P ≤ 0.05

At the one year post-operative period, 120 eyes comprised the FLACS group and 265 eyes for the CP group. Attrition rate was at 59.46% for FLACS and 39.64% for CP. MRSE of the 2 groups were analyzed. Both groups had statistically similar percentages of patients per half diopter range of MRSE (Table 2).

**Table 2:** Post-operative Manifest Refraction Spherical Equivalent at 1 Year

	% (No. of patients)		*P* Value
	FLACS (*n* = 120)	CP (*n* = 265)	
≤0.50 D	65.83% (79)	72.45% (192)	0.09
0.51 D to 1.00 D	24.17% (29)	22.26% (59)	
1.01 D to 1.50 D	9.17% (11)	3.40% (9)	
1.51 D to 2.00 D	0.83% (1)	1.89% (5)	

*CP =* conventional phacoemulsification; *D =* diopter; *FLACS =* femtosecond laser-assisted cataract surgery

^*^P ≤ 0.05

**Fig. 7:**
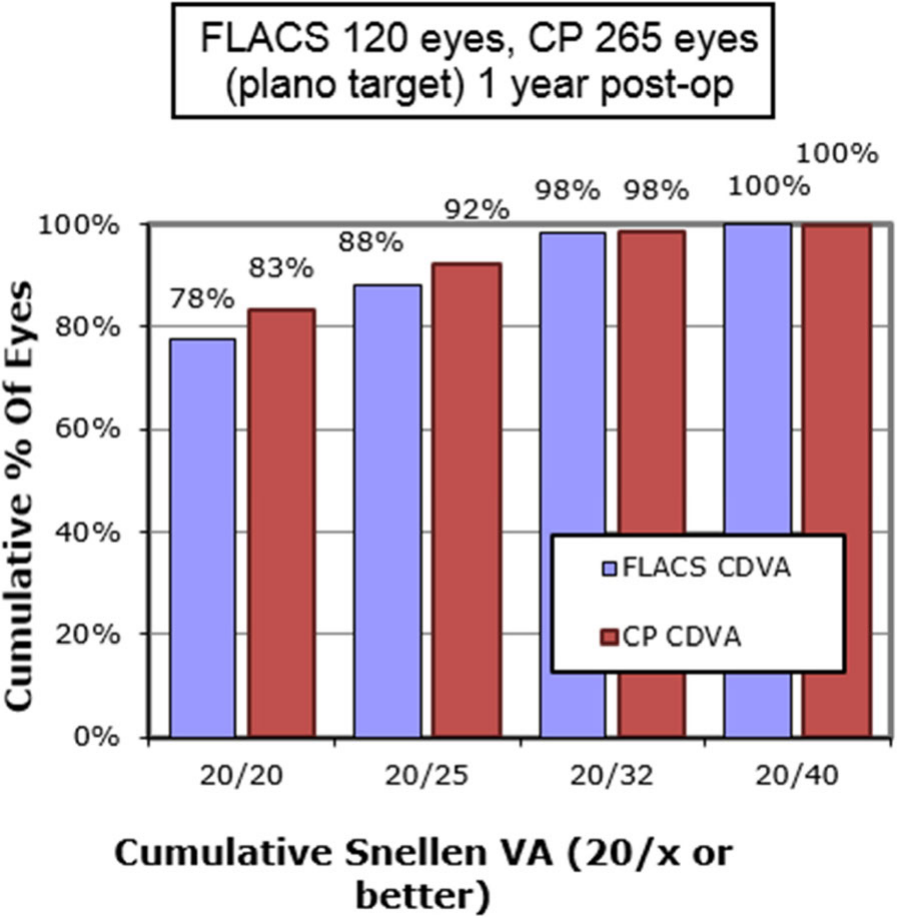
Post-operative corrected distance visual acuity at 1 year per Snellen Chart

Subgroup analysis was performed based on the type of IOL. All IOLs and their respective subgroups (monofocal, monofocal toric, multifocal, multifocal toric, accommodating) were analyzed for MRSE (Table 3), Mean Refractive Cylinder (Table 4), UDVA (Table 5) and CDVA (Table 6). MRSE and UDVA between the two groups were almost similar on the pre-operative and routine follow-ups. No significant difference between the FLACS and CP groups was noted in the percentage of patients who achieved a CDVA of 20/25 or better (Fig. 7). The multifocal IOLs and accommodating IOLs were also analyzed based on UNVA and DCNVA (Table 7). Overall there was no statistically significant trend seen even with the IOL subgroup analysis.

**Table 3:** Mean Refractive Spherical Equivalent (logMAR) and Mean Absolute Error (MAE)

IOL Grouping	FLACS			CP			*P* Value
	n	MRSE ± SD	MAE	n	MRSE ± SD	MAE	
Overall MRSE							
1 Day	286	−0.17 ± 0.71	0.17	371	− 0.13 ± 0.61	0.13	0.4383
1 Week	283	−0.24 ± 0.53	0.24	417	− 0.21 ± 0.50	0.21	0.4473
1 Month	239	−0.21 ± 0.53	0.21	367	− 0.22 ± 0.52	0.22	0.8185
3 Months	191	−0.22 ± 0.57	0.22	315	− 0.23 ± 0.52	0.23	0.8399
6 Months	164	−0.20 ± 0.66	0.20	268	− 0.24 ± 0.57	0.24	0.5057
1 Year	120	−0.16 ± 0.58	0.16	265	− 0.20 ± 0.52	0.20	0.5007
Monofocal MRSE (target plano)							
1 Day	53	−0.21 ± 0.63	0.21	45	− 0.03 ± 0.78	0.03	0.5676
1 Week	57	− 0.27 ± 0.46	0.27	65	−0.17 ± 0.56	0.17	0.2874
1 Month	45	−0.23 ± 0.34	0.23	53	−0.13 ± 0.56	0.13	0.2986
3 Months	28	−0.25 ± 0.35	0.25	40	−0.08 ± 0.43	0.08	0.0886
6 Months	24	−0.12 ± 0.78	0.12	33	−0.05 ± 0.50	0.05	0.6815
1 Year	25	−0.06 ± 0.53	0.06	32	−0.01 ± 0.56	0.01	0.7334
Monofocal Toric MRSE (target plano)							
1 Day	57	−0.07 ± 0.59	0.07	62	−0.06 ± 0.53	0.06	0.9226
1 Week	59	−0.11 ± 0.70	0.11	59	−0.05 ± 0.64	0.05	0.6280
1 Month	50	−0.12 ± 0.63	0.12	57	−0.11 ± 0.45	0.11	0.9243
3 Months	40	−0.20 ± 0.71	0.20	43	−0.08 ± 0.52	0.08	0.3800
6 Months	36	−0.16 ± 0.71	0.16	35	−0.06 ± 0.45	0.06	0.4822
1 Year	27	−0.09 ± 0.74	0.09	35	−0.03 ± 0.49	0.03	0.7028
Multifocal MRSE (target plano)							
1 Day	77	−0.18 ± 0.70	0.18	101	−0.22 ± 0.56	0.22	0.6724
1 Week	77	−0.22 ± 0.49	0.22	98	−0.22 ± 0.52	0.22	> 0.9999
1 Month	60	−0.19 ± 0.52	0.19	85	− 0.18 ± 0.54	0.18	0.9114
3 Months	42	−0.11 ± 0.47	0.11	69	− 0.07 ± 0.55	0.07	0.6958
6 Months	31	−0.12 ± 0.59	0.12	51	− 0.17 ± 0.58	0.17	0.7078
1 Year	21	−0.17 ± 0.47	0.17	48	− 0.02 ± 0.43	0.02	0.1994
Multifocal Toric MRSE (target plano)							
1 Day	49	−0.38 ± 0.50	0.38	66	−0.25 ± 0.44	0.25	0.1422
1 Week	43	−0.17 ± 0.92	0.17	58	−0.15 ± 0.56	0.15	0.0328^*^
1 Month	36	−0.10 ± 0.50	0.10	53	−0.26 ± 0.48	0.26	0.1327
3 Months	27	−0.25 ± 0.40	0.25	48	−0.24 ± 0.45	0.24	0.9238
6 Months	24	0.00 ± 0.68	0.00	48	−0.16 ± 0.50	0.16	0.2616
1 Year	25	0.00 ± 0.66	0.00	52	−0.19 ± 0.45	0.19	0.1422
Accommodating MRSE (target −0.50)							
1 Day	41	−0.33 ± 0.72	0.17	116	−0.22 ± 0.53	0.28	0.3023
1 Week	40	−0.49 ± 0.60	0.01	118	−0.32 ± 0.45	0.18	0.0607
1 Month	35	−0.43 ± 0.56	0.07	113	−0.35 ± 0.50	0.15	0.4229
3 Months	31	−0.55 ± 0.69	0.05	110	−0.45 ± 0.49	0.05	0.3636
6 Months	26	−0.61 ± 0.59	0.11	95	−0.50 ± 0.56	0.00	0.3820
1 Year	20	−0.46 ± 0.58	0.04	95	−0.46 ± 0.50	0.04	> 0.9999

*CP =* conventional phacoemulsification; *FLACS =* femtosecond laser-assisted cataract surgery; *IOL =* intraocular lens; *logMAR =* logarithm of the minimum angle of resolution; *MAE =* mean absolute error; *MRSE =* manifest refraction spherical equivalent; *SD =* standard deviation

^*^P < 0.05

**Table 4:** Manifest Refractive Cylinder (Diopter)

	FLACS	CP	P value
1 Month	− 0.75 ± 0.46	−0.72 ± 0.44	0.47
3 Months	− 0.70 ± 0.40	−0.74 ± 0.45	0.32
6 Months	− 0.75 ± 0.48	−0.74 ± 0.43	0.83
1 Year	− 0.75 ± 0.50	−0.72 ± 0.44	0.64

*FLACS =* femtosecond laser-assisted cataract surgery; *CP =* conventional phacoemulsification

**Table 5:** Uncorrected Distance Visual Acuity for All Intraocular Lenses (logMAR)

IOL Grouping	FLACS		CP		*P* Value
	n	Mean ± SD	n	Mean ± SD	
Overall UDVA					
1 Day	293	0.17 ± 0.17 (20/25)	427	0.15 ± 0.19 (20/25)	0.15
1 Week	282	0.13 ± 0.14 (20/25)	420	0.11 ± 0.14 (20/25)	0.06
1 Month	244	0.13 ± 0.15 (20/25)	369	0.11 ± 0.15 (20/25)	0.11
3 Months	191	0.12 ± 0.13 (20/25)	315	0.12 ± 0.14 (20/25)	1.00
6 Months	162	0.12 ± 0.14 (20/25)	268	0.10 ± 0.13 (20/25)	0.13
1 Year	120	0.12 ± 0.13 (20/25)	265	0.11 ± 0.13 (20/25)	0.48
Monofocal UDVA					
1 Day	59	0.15 ± 0.15 (20/25)	67	0.16 ± 0.22 (20/25)	0.08
1 Week	58	0.13 ± 0.14 (20/25)	65	0.13 ± 0.19 (20/25)	1.00
1 Month	47	0.15 ± 0.16 (20/25)	54	0.13 ± 0.17 (20/25)	0.55
3 Months	28	0.13 ± 0.15 (20/25)	39	0.14 ± 0.15 (20/25)	0.08
6 Months	23	0.13 ± 0.13 (20/25)	34	0.14 ± 0.16 (20/25)	0.80
1 Year	25	0.12 ± 0.13 (20/25)	32	0.12 ± 0.13 (20/25)	1.00
Monofocal Toric UDVA					
1 Day	57	0.15 ± 0.14 (20/25)	63	0.13 ± 0.12 (20/25)	0.40
1 Week	59	0.21 ± 0.16 (20/32)	66	0.15 ± 0.20 (20/25)	0.07
1 Month	51	0.18 ± 0.16 (20/25)	58	0.12 ± 0.18 (20/25)	0.07
3 Months	39	0.16 ± 0.13 (20/25)	43	0.14 ± 0.15 (20/25)	0.52
6 Months	37	0.16 ± 0.14 (20/25)	34	0.10 ± 0.12 (20/25)	0.06
1 Year	27	0.16 ± 0.15 (20/25)	34	0.15 ± 0.13 (20/25)	0.78
Multifocal UDVA					
1 Day	78	0.18 ± 0.19 (20/25)	101	0.15 ± 0.20 (20/25)	0.31
1 Week	75	0.13 ± 0.15 (20/25)	99	0.11 ± 0.13 (20/25)	0.35
1 Month	62	0.11 ± 0.14 (20/25)	85	0.12 ± 0.15 (20/25)	0.68
3 Months	43	0.10 ± 0.12 (20/25)	70	0.13 ± 0.17 (20/25)	0.31
6 Months	30	0.11 ± 0.15 (20/25)	51	0.12 ± 0.14 (20/25)	0.76
1 Year	21	0.10 ± 0.12 (20/25)	50	0.11 ± 0.12 (20/25)	0.75
Multifocal Toric UDVA					
1 Day	49	0.13 ± 0.12 (20/25)	67	0.15 ± 0.17 (20/25)	0.48
1 Week	43	0.20 ± 0.24 (20/32)	69	0.18 ± 0.18 (20/25)	0.62
1 Month	36	0.13 ± 0.14 (20/25)	53	0.15 ± 0.14 (20/25)	0.51
3 Months	27	0.10 ± 0.11 (20/25)	48	0.17 ± 0.14 (20/25)	0.03^*^
6 Months	24	0.18 ± 0.15 (20/25)	48	0.13 ± 0.13 (20/25)	0.15
1 Year	25	0.10 ± 0.14 (20/25)	52	0.16 ± 0.14 (20/25)	0.08
Accommodating UDVA					
1 Day	41	0.14 ± 0.14 (20/25)	116	0.14 ± 0.16 (20/25)	1.00
1 Week	40	0.15 ± 0.15 (20/25)	118	0.08 ± 0.12 (20/20)	0.00^*^
1 Month	35	0.12 ± 0.14 (20/25)	113	0.07 ± 0.11 (20/20)	0.03^*^
3 Months	31	0.14 ± 0.15 (20/25)	113	0.00 ± 0.01 (20/20)	0.00^*^
6 Months	25	0.12 ± 0.12 (20/25)	95	0.08 ± 0.12 (20/20)	0.14
1 Year	20	0.12 ± 0.14 (20/25)	94	0.07 ± 0.10 (20/20)	0.06

*CP =* conventional phacoemulsification; *FLACS =* femtosecond laser-assisted cataract surgery; *IOL =* intraocular lens; *logMAR =* logarithm of the minimum angle of resolution; *SD =* standard deviation; *UDVA =* uncorrected distance visual acuity

^*^P ≤ 0.05

**Table 6:** Corrected Distance Visual Acuity for All Intraocular Lenses (logMAR)

IOL Grouping	FLACS		CP		*P* Value
	n	Mean ± SD	n	Mean ± SD	
Overall CDVA					
1 Day	285	0.09 ± 0.14 (20/20)	363	0.07 ± 0.13 (20/20)	0.06
1 Week	278	0.05 ± 0.09 (20/20)	411	0.04 ± 0.08 (20/20)	0.13
1 Month	236	0.05 ± 0.09 (20/20)	357	0.03 ± 0.07 (20/20)	0.00^*^
3 Months	191	0.03 ± 0.08 (20/20)	312	0.03 ± 0.07 (20/20)	1.00
6 Months	161	0.04 ± 0.08 (20/20)	266	0.03 ± 0.07 (20/20)	0.18
1 Year	120	0.03 ± 0.07 (20/20)	265	0.02 ± 0.06 (20/20)	0.15
Monofocal CDVA					
1 Day	53	0.08 ± 0.12 (20/20)	43	0.06 ± 0.11 (20/20)	0.40
1 Week	54	0.05 ± 0.09 (20/20)	64	0.04 ± 0.09 (20/20)	0.55
1 Month	43	0.07 ± 0.11 (20/20)	52	0.03 ± 0.09 (20/20)	0.05
3 Months	28	0.07 ± 0.12 (20/20)	40	0.03 ± 0.06 (20/20)	0.07
6 Months	22	0.04 ± 0.08 (20/20)	33	0.04 ± 0.08 (20/20)	1.00
1 Year	24	0.04 ± 0.08 (20/20)	32	0.01 ± 0.04 (20/20)	0.07
Monofocal Toric CDVA					
1 Day	57	0.05 ± 0.10 (20/20)	60	0.05 ± 0.09 (20/20)	1.00
1 Week	59	0.11 ± 0.16 (20/25)	55	0.08 ± 0.13 (20/20)	0.28
1 Month	50	0.06 ± 0.12 (20/20)	55	0.05 ± 0.09 (20/20)	0.63
3 Months	40	0.04 ± 0.09 (20/20)	41	0.06 ± 0.10 (20/20)	0.00^*^
6 Months	36	0.04 ± 0.07 (20/20)	35	0.05 ± 0.08 (20/20)	0.58
1 Year	27	0.03 ± 0.07 (20/20)	35	0.07 ± 0.09 (20/20)	0.06
Multifocal CDVA					
1 Day	77	0.10 ± 0.14 (20/25)	101	0.08 ± 0.15 (20/20)	0.37
1 Week	75	0.05 ± 0.10 (20/20)	97	0.04 ± 0.08 (20/20)	0.47
1 Month	59	0.05 ± 0.09 (20/20)	83	0.03 ± 0.06 (20/20)	0.11
3 Months	43	0.03 ± 0.06 (20/20)	69	0.03 ± 0.07 (20/20)	1.00
6 Months	31	0.04 ± 0.09 (20/20)	49	0.03 ± 0.07 (20/20)	0.58
1 Year	21	0.02 ± 0.07 (20/20)	48	0.03 ± 0.07 (20/20)	0.59
Multifocal Toric CDVA					
1 Day	49	0.04 ± 0.11 (20/20)	66	0.06 ± 0.11 (20/20)	0.34
1 Week	43	0.10 ± 0.16 (20/25)	58	0.09 ± 0.14 (20/20)	0.74
1 Month	36	0.04 ± 0.07 (20/20)	53	0.05 ± 0.09 (20/20)	0.58
3 Months	27	0.03 ± 0.08 (20/20)	48	0.04 ± 0.08 (20/20)	0.60
6 Months	24	0.03 ± 0.09 (20/20)	48	0.04 ± 0.09 (20/20)	0.66
1 Year	25	0.02 ± 0.14 (20/20)	52	0.03 ± 0.07 (20/20)	0.68
Accommodating CDVA					
1 Day	40	0.02 ± 0.04 (20/20)	115	0.05 ± 0.12 (20/20)	0.12
1 Week	40	0.04 ± 0.08 (20/20)	118	0.02 ± 0.05 (20/20)	0.07
1 Month	35	0.01 ± 0.03 (20/20)	113	0.01 ± 0.03 (20/20)	1.00
3 Months	30	0.00 ± 0.02 (20/20)	110	0.00 ± 0.02 (20/20)	1.00
6 Months	25	0.02 ± 0.06 (20/20)	95	0.00 ± 0.02 (20/20)	0.01^*^
1 Year	20	0.01 ± 0.05 (20/20)	95	0.00 ± 0.03 (20/20)	0.24

*CDVA =* corrected distance visual acuity; *CP =* conventional phacoemulsification; *FLACS =* femtosecond laser-assisted cataract surgery; *IOL =* intraocular lens; *logMAR =* logarithm of the minimum angle of resolution; *SD =* standard deviation

^*^P ≤ 0.05

**Table 7:** Near Visual Acuity for Multifocals and Accommodating Intraocular Lenses (logMAR)

IOL Grouping	FLACS		CP		*P* Value
	n	Mean ± SD	n	Mean ± SD	
Multifocal UNVA					
1 Day	78	0.18 ± 0.20 (J3)	81	0.13 ± 0.16 (J2)	0.08
1 Week	74	0.10 ± 0.15 (J2)	82	0.10 ± 0.12 (J2)	1.00
1 Month	60	0.11 ± 0.15 (J2)	68	0.08 ± 0.08 (J1)	0.15
3 Months	42	0.09 ± 0.14 (J1)	57	0.11 ± 0.12 (J2)	0.45
6 Months	30	0.08 ± 0.15 (J1)	41	0.09 ± 0.10 (J1)	0.74
1 Year	21	0.10 ± 0.11 (J2)	40	0.12 ± 0.12 (J2)	0.53
Multifocal DCNVA					
1 Day	77	0.16 ± 0.19 (J2)	79	0.14 ± 0.20 (J2)	0.52
1 Week	74	0.07 ± 0.12 (J1)	74	0.10 ± 0.13 (J2)	0.15
1 Month	57	0.08 ± 0.12 (J1)	59	0.06 ± 0.09 (J1)	0.31
3 Months	42	0.05 ± 0.08 (J1)	53	0.10 ± 0.12 (J2)	0.02^*^
6 Months	30	0.06 ± 0.10 (J1)	36	0.07 ± 0.11 (J1)	0.70
1 Year	21	0.09 ± 0.11 (J1)	38	0.09 ± 0.14 (J1)	1.00
Accommodating UNVA					
1 Day	39	0.23 ± 0.14 (J4)	115	0.17 ± 0.12 (J2)	0.01
1 Week	39	0.29 ± 0.16 (J4)	112	0.23 ± 0.14 (J4)	0.03
1 Month	34	0.22 ± 0.15 (J4)	111	0.15 ± 0.11 (J2)	0.00^*^
3 Months	30	0.18 ± 0.14 (J3)	110	0.14 ± 0.11 (J2)	0.10
6 Months	25	0.15 ± 0.11 (J2)	94	0.14 ± 0.10 (J2)	0.66
1 Year	17	0.19 ± 0.16 (J3)	93	0.13 ± 0.13 (J2)	0.09
Accommodating DCNVA					
1 Day	30	0.27 ± 0.15 (J4)	115	0.24 ± 0.14 (J4)	0.30
1 Week	22	0.33 ± 0.19 (J5)	112	0.22 ± 0.14 (J4)	0.00^*^
1 Month	26	0.25 ± 0.12 (J4)	111	0.19 ± 0.12 (J3)	0.00^*^
3 Months	22	0.21 ± 0.11 (J4)	105	0.17 ± 0.12 (J2)	0.15
6 Months	19	0.17 ± 0.11 (J2)	91	0.19 ± 0.12 (J3)	0.50
1 Year	13	0.25 ± 0.12 (J4)	91	0.19 ± 0.12 (J3)	0.09

*CP =* conventional phacoemulsification; *DCNVA =* distance-corrected near visual acuity; *FLACS =* femtosecond laser-assisted cataract surgery; *IOL =* intraocular lens; *logMAR =* logarithm of the minimum angle of resolution; *SD =* standard deviation; *UNVA =* uncorrected near visual acuity

^*^P ≤ 0.05

Intraoperative parameters that were unique to FLACS included capsulotomy quality and incidence of suction break (Table 8). In one patient with grade 4 white cataract, an anterior capsular tear occurred while performing the femtosecond laser treatment. The tear was visualized and removal of nucleus and cortical material proceeded with care. The other two anterior capsular tears occurred after manually completing an incomplete laser-assisted capsulotomy and a residual bridge. However, all capsular tears did not extend posteriorly and the IOL was placed in the bag for each case with no other complications. The rest of the capsulotomies that were not freely floating were successfully completed manually (Fig. 9). Suction break occurred in 2 instances due to a narrow palpebral fissure and patients squeezing the suction slip (Fig. 10).

**Table 8:** Femtosecond Laser Treatment Complications

	FLACS (*n* = 296) No. of patients (%)
Capsulotomy	
Adhesion	26 (8.78%)
Bridge	3 (1.01%)
Incomplete	2 (0.68%)
Anterior Capsular Tear	3 (1.01%)
Posterior Capsular Tear	0 (0%)
Suction Break	2 (0.68%)

The mean values of cumulative dissipated energy (CDE) during phacoemulsification for the FLACS group were lower in all cataract grades compared to the CP group. The CDE was significantly lower with grade 4 cataracts in the FLACS group (Table 9).

**Table 9:** Cumulative Dissipated Energy During Phacoemulsification

LOCS III Grade	FLACS		CP		*P* Value
	n	CDE (percent-seconds) Mean ± SD	n	CDE (percent-seconds) Mean ± SD	
NO1	25	6.97 ± 5.74	36	7.59 ± 6.42	0.70
NO2	136	14.23 ± 8.86	239	15.34 ± 10.33	0.29
NO3	79	26.86 ± 10.73	111	27.02 ± 9.78	0.92
NO4	56	29.02 ± 16.07	53	35.69 ± 18.30	0.05^*^

*CDE =* cumulative dissipated energy; *CP =* conventional phacoemulsification; *FLACS =* femtosecond laser-assisted cataract surgery; *LOCS III =* Lens Opacities Classification System III; *NO =* nuclear opalescence; *SD =* standard deviation

^*^P ≤ 0.05

Post-operative mean grading for corneal edema, aqueous cells, flare, and endothelial cell loss on specular microscopy were compared in both groups. Post-operative central corneal edema at 1 day, aqueous cells and flare at 1 week and endothelial cell loss at 1 month for FLACS were found to be significantly lower statistically compared to the CP group (Table 10).

**Table 10:** Post-operative Corneal Edema, Cells and Flare, and Endothelial Cell Loss

	FLACS		CP		*P* Value
	n	Mean ± SD	n	Mean ± SD	
Corneal Edema^a^ (Central)					
1 Day	263	0.65 ± 0.67	196	0.78 ± 0.73	0.05^*^
1 Week	170	0.05 ± 0.21	88	0.07 ± 0.24	0.49
Corneal Edema^a^ (Peripheral)					
1 Day	142	1.03 ± 0.77	79	1.02 ± 0.62	0.92
1 Week	85	0.14 ± 0.36	44	0.13 ± 0.34	0.88
Aqueous Cells and Flare^b^					
1 Day	140	0.96 ± 0.59	72	1.04 ± 0.54	0.34
1 Week	80	0.03 ± 0.16	42	0.16 ± 0.40	0.01^*^
ECD (cells/mm^2^)					
Baseline	283	2602.24 ± 497.30	353	2631.28 ± 387.42	0.41
1 Month	189	2443.67 ± 658.78	69	2377.26 ± 459.82	0.66
EC Loss	189	175.23 ± 649.54	69	351.52 ± 454.21	0.04^*^

*EC =* endothelial cell; *ECD =* endothelial cell density; *CP =* conventional phacoemulsification; *FLACS =* femtosecond laser-assisted cataract surgery; *SD =* standard deviation

^a^Grading of corneal edema: 0 no trace; +1 Trace; +2 Mild; +3 Moderate; +4 Severe

^b^Standardization of Uveitis Nomenclature (SUN) Working Group Classification for anterior chamber inflammation

^*^P-value < 0.05

## Discussion

The last significant technological revolution in cataract surgery was the introduction of phacoemulsification in the early 1990s. Back then, there was resistance to change because surgeons were already achieving consistently good outcomes from extracapsular cataract surgery with minimal investment in equipment. It took more than a decade of developing new instruments, foldable IOLs and a critical mass of instructors to establish phacoemulsification as the primary procedure of choice for surgeons. We are again at an important point of evolution and change. The femtosecond laser can perform critical steps in cataract surgery that can make the entire procedure more consistent in quality and usher in a new generation of innovations. However, it still needs to be proven whether femtosecond laser-assisted cataract surgery (FLACS) is as safe and can produce better outcomes than the current gold-standard, which is conventional phacoemulsification (CP). In this retrospective review, the authors present their clinical outcomes in order to help answer this important question.

FLACS was first introduced in our practice as an alternative to CP in 2013. The conversion rate that initial year was at 58%, with gradual increases in 2014 and 2015 to 60% and 66%, respectively. Of those patients who underwent CP over FLACS, 98% cited cost as the main reason they did not choose FLACS. Of the remaining 2% of patients, we disqualified them from undergoing FLACS because they had small interpalpebral apertures that may cause problems with docking, poorly dilating pupil that did not reach the size of the intended capsulotomy, and shallow anterior chamber at risk for IOP rise.

In our study, we decided to do a subgroup analysis of refractive and visual outcomes based on the type of IOL to remove any bias related to IOL power targeting between lenses and get a clearer picture if some types of lenses benefit more from the precision of FLACS. We found no significant difference in outcomes up to one year follow up between FLACS and CP, so we benchmarked our results with studies in the literature to confirm our findings.

Filkorn et al. divided their population based on axial length and found that the FLACS group had a significantly lower mean absolute error in spherical equivalent refraction, with the greatest difference in short and long eyes [35]. Ewe et al. reported that in their CP group, a higher percentage of eyes were within 0.50 D of error from target refraction and the difference in their study was significant [36]. Conrad-Hengerer et al. investigated manifest refraction and CDVA deviation from target refraction, and refractive stability up to six months post-operatively [37]. FLACS had better refractive results up to 1 week hence stabilized earlier but did not differ significantly compared to CP afterwards [37]. They determined that FLACS is safe and precise, however, only enhanced visual outcomes minimally with a significant difference in refractive deviation but had no clinical relevance [37]. In a study by Miháltz et al., the FLACS and CP groups were compared based on sphere, cylinder, UDVA and CDVA, with the two groups only differing in the method of capsulorhexis [38]. There was also no statistically significant difference at 6 months after surgery. In our study, FLACS had no statistically significant difference compared to CP for refractive and visual outcomes up to one year follow-up, except for a few time points, which proved temporary. In terms of residual refractive astigmatism (MRSE – Table 3 and Mean Refractive Cylinder – Table 4), no significant difference was noted between FLACS and CP in our single surgeon study, suggesting that the effect is similar whether the corneal incision was made with the laser or manually.

No trend emerged from the analysis of MRSE, UDVA, and CDVA for the different types of IOLs. Overall, manually performed cataract removal for standard cases in the hands of an experienced surgeon provided similar levels of visual acuity results. FLACS demonstrated a comparable visual outcome with CP but did not establish a clinical visual advantage over it [14, 35–38]. Clinical evidence at this time, including our study, supports this finding and we therefore concur with this observation.

The concept of FLACS and the higher cost associated with it leads the surgeon to expect completely free floating, perfectly symmetrical and consistently sized capsulotomies in each and every case the laser is applied. This is achievable in most cases, but unique types of non-completion lead us to categorize these occurrences as complications. Even in stricter terms, it can be interpreted as non-fulfillment of the expectations of tasks the laser had to perform. These so-called complications uniquely encountered in FLACS cases include non-free floating capsulotomies, anterior capsular tears, and suction breaks. Our anterior capsular tear rate was low and we had zero incidence of posterior capsular extension in the FLACS group. Reports from other studies ranged from 0 to 5.3% for the anterior capsular tear rate and a maximum of 50% incidence of extension to the posterior capsule when an anterior capsular tear occurred [10, 20–22, 25, 26, 39]. In our study, an anterior capsular tear occurred during FLACS of a patient with grade 4 white cataract. The egress of milky fluid can pose a challenge during capsulotomy even though the study by Conrad-Hengerer et al. reported that FLACS is safe and technically feasible in intumescent white cataracts, stating that capsule tears occur less frequently in FLACS compared to CP [27]. Complications listed by other studies but not encountered by the surgeon in the charts reviewed except for subconjunctival hemorrhage include corneal haze affecting the surgical view, laser-induced miosis, anterior capsulotomy tag, posterior capsular rupture, dropped nucleus, capsular block syndrome, endothelial damage due to laser cuts within the endothelial layer, and docking attempts [10, 20–22, 25, 26, 39]. The FLACS complications noted in our study did not decrease in incidence over the 3 years hence were not attributable to inadequate surgeon experience and were not correlated to the grade of the cataract.

A suction force is applied on the eye to stabilize its position as the laser is firing. Our definition of suction break is non-completion of the femtosecond laser procedures of capsulotomy, lens fragmentation and corneal incisions because the attachment of the eye to the patient interface of the laser was released prematurely. We had 0.68% incidence that fell within the range of other studies varying from 0 to 2.5% [20–22, 39]. The suction breaks were attributed to small palpebral fissures and patients squeezing the clip until the suction force was broken. Other main risk factors include inadvertent eye or head movement of the patient, improper docking, and loose conjunctiva around the limbus [21]. The most important factors to prevent suction breaks are precise patient interface placement and sufficient preoperative topical anesthesia [21]. A suction break is not a major concern because whatever steps were not performed by the femtosecond laser can be completed manually by the surgeon.

Perhaps the most significant benefit a patient receives when having a FLACS procedure is less ultrasonic energy delivered into the anterior chamber because the cataractous lens has been pre-cut. Ultrasonic energy during phacoemulsification can be quantified either by cumulative dissipated energy (CDE) or effective phacoemulsification time (EPT). CDE is effective phacoemulsification time (EPT) in minutes divided by 100 while EPT is phacoemulsification time in seconds multiplied by the average phacoemulsification power in percentage [40]. In the literature, all authors agree that FLACS significantly reduced EPT compared to CP [9, 14, 15, 17, 18]. In our study, the CDE values for FLACS were all lower compared to CP per cataract grade but the statistically significant difference was noted for grade 4 cataracts. Further reduction of EPT was proposed by Abell et al. through optimization of lens fragmentation patterns and surgical technique, with zero EPT as a possibility [14]. FLACS can be most beneficial for mature cataracts, those with zonular compromise and with compromised corneas since decreased EPT will be deemed crucial for these [36]. No study has been published on the advantage of FLACS over CP for such cases [36].

Less phacoemulsification ultrasonic energy in FLACS ideally translates to less corneal edema, less anterior chamber inflammation and a reduced loss of endothelial cell count. Central corneal edema for FLACS in our study was statistically lower post-operatively at 1 day but the difference was not significant by 1 week. Peripheral corneal edema in FLACS was higher than CP due to higher laser energy delivered to create the corneal main incision but the difference with CP was not statistically significant. Abell et al. had similar central corneal edema findings, with statistically significant reduction in corneal edema for FLACS one day post-operatively but no difference at three weeks [18].

Anterior chamber cells and flare were significantly lower with FLACS at one week after surgery. This finding was consistent with Abell et al. and was attributed to decreased EPT in FLACS [17]. Endothelial cell loss was statistically significantly lower for FLACS compared to CP one month after surgery. Conrad-Hengerer et al. studied the mean endothelial cell loss of FLACS compared to CP up to 3 months follow-up and found that FLACS did not add to the endothelial damage and may be favorable in cases of low preoperative endothelial cell values [19].

The present study has several limitations. First is the absence of randomization due to its retrospective nature. Second, the study highlights the real-world scenario wherein the ability or willingness of the patient to pay out of pocket to have femtosecond laser assisted cataract surgery was the most common deciding factor, not a medical indication, for selection of femtosecond laser versus manual phacoemulsification. Third is the preference of the surgeon to use toric IOL implantation rather than using the femtosecond laser limbal relaxing incision (LRI) option. The unused benefit of femtosecond laser LRI could have affected refractive outcomes but in clinical practice, the surgeon has the prerogative to use the features of a technology in that they are most comfortable with.

Overall, FLACS has two clear benefits. First is delivery of lower phacoemulsification energy resulting in reduced endothelial cell loss and less anterior chamber inflammation. Second is providing a consistent capsulotomy that can help an inexperienced surgeon with this crucial step but can also help any surgeon when the anterior capsule opening needs to be consistently sized for specific intraocular lenses. With these benefits, FLACS may be particularly beneficial in complex cases with greater risk for corneal endothelial decompensation. These include patients diagnosed with hypermature cataracts, Fuchs dystrophy or those with highly brunescent nuclei. Cases with loose zonular fibers may also benefit from the automated capsulotomy of femtosecond laser as it spares the zonules from the additional stress of manually tugging on the anterior capsule.

At this time, these advantages have not translated to statistically better refractive and visual outcomes compared to conventional phacoemulsification. Reports continue to be published in the literature as experience grows. Admittedly, cost is an issue and perhaps the main hindrance to widespread adaptation of this technology. However, we see no disadvantage of FLACS over CP whether from the literature or based on our experience. We recommend that the option of FLACS be explained objectively to each patient and allow them to decide whether the benefits of FLACS outweigh their cost considerations.

## Conclusion

Femtosecond laser-assisted cataract surgery and conventional phacoemulsification had similar refractive and visual outcomes. Use of femtosecond laser in cataract surgery was efficient and safe. Femtosecond laser-assisted cataract surgery reduced the phacoemulsification energy, decreased the amount of corneal endothelial cell loss and resulted in lesser anterior chamber inflammation compared with conventional phacoemulsification.

## Abbreviations

CDVA: Corrected distance visual acuity
CP: Conventional phacoemulsification
D: Diopter
DCNVA: Distance-corrected near visual acuity
EC: Endothelial cell
ECD: Endothelial cell density
FLACS: Femtosecond laser-assisted cataract surgery
IOL: Intraocular lens
LOCS III: Lens Opacities Classification System III
logMAR: logarithm of the minimum angle of resolution
MRSE: Manifest refraction spherical equivalent
NO: Nuclear opalescence
SD: Standard deviation
SUN: Standardization of Uveitis Nomenclature
UDVA: Uncorrected distance visual acuity
UNVA: Uncorrected near visual acuity
